# Linking CD1-Restricted T Cells With Autoimmunity and Dyslipidemia: Lipid Levels Matter

**DOI:** 10.3389/fimmu.2018.01616

**Published:** 2018-07-16

**Authors:** Sreya Bagchi, Samantha Genardi, Chyung-Ru Wang

**Affiliations:** Department of Microbiology and Immunology, Northwestern University, Chicago, IL, United States

**Keywords:** CD1, dyslipidemia, antigen presentation, autoreactive T cells, natural killer T cells, animal models

## Abstract

Dyslipidemia, or altered blood lipid content, is a risk factor for developing cardiovascular disease. Furthermore, several autoimmune diseases, including systemic lupus erythematosus, psoriasis, diabetes, and rheumatoid arthritis, are correlated highly with dyslipidemia. One common thread between both autoimmune diseases and altered lipid levels is the presence of inflammation, suggesting that the immune system might act as the link between these related pathologies. Deciphering the role of innate and adaptive immune responses in autoimmune diseases and, more recently, obesity-related inflammation, have been active areas of research. The broad picture suggests that antigen-presenting molecules, which present self-peptides to autoreactive T cells, can result in either aggravation or amelioration of inflammation. However, very little is known about the role of self-lipid reactive T cells in dyslipidemia-associated autoimmune events. Given that a range of autoimmune diseases are linked to aberrant lipid profiles and a majority of lipid-specific T cells are reactive to self-antigens, it is important to examine the role of these T cells in dyslipidemia-related autoimmune ailments and determine if dysregulation of these T cells can be drivers of autoimmune conditions. CD1 molecules present lipids to T cells and are divided into two groups based on sequence homology. To date, most of the information available on lipid-reactive T cells comes from the study of group 2 CD1d-restricted natural killer T (NKT) cells while T cells reactive to group 1 CD1 molecules remain understudied, despite their higher abundance in humans compared to NKT cells. This review evaluates the mechanisms by which CD1-reactive, self-lipid specific T cells contribute to dyslipidemia-associated autoimmune disease progression or amelioration by examining available literature on NKT cells and highlighting recent progress made on the study of group 1 CD1-restricted T cells.

## Introduction

Over the years, it has become apparent that a range of rheumatological and dermatological autoimmune diseases like systemic lupus erythematosus (SLE) and psoriasis are associated with dyslipidemia (1, 2). In most cases, dyslipidemia in autoimmune diseases is characterized by altered serum cholesterol, triglyceride (TG), low-density lipoprotein (LDL), and high-density lipoprotein (HDL) levels (3–5). In this review, the words dyslipidemia and lipid abnormalities will be used interchangeably, while hyperlipidemia will be used to refer to cases in which an increase in cholesterol, TGs, and LDL was observed.

Atherogenic lipid profiles, characterized by increased serum cholesterol and TGs, have been observed up to 10 years prior to diagnosis of rheumatoid arthritis (6), while hypercholesterolemia in SLE patients has only been reported after onset of disease (7, 8). Overall, these data indicate that dyslipidemia might be linked to autoimmune disease. However, whether dyslipidemia acts as a potential trigger for the initiation of autoimmune diseases has not been investigated in-depth. It is known that inflammation plays a key role in autoimmune diseases (9–12). Several studies have shown that obesity gives rise to low-grade chronic inflammation and the incidence and severity of autoimmune diseases, particularly, rheumatoid arthritis and psoriasis, are increased in obese patients compared to population controls (13). These data suggest that metabolic inputs influence inflammatory outputs (14). In obese individuals, for example, adipocytes release pro-inflammatory cytokines and adipokines, like TNF-α, IL-1β, and leptin, which activate both innate and adaptive arms of the immune system (14, 15). Therefore, in addition to genetic and environmental stress, dyslipidemia-induced chronic inflammation could be a driver of autoimmune diseases, suggesting an intricate interplay between lipid metabolism, activation of the immune system, and subsequent development of autoimmune diseases.

**Figure 1:**
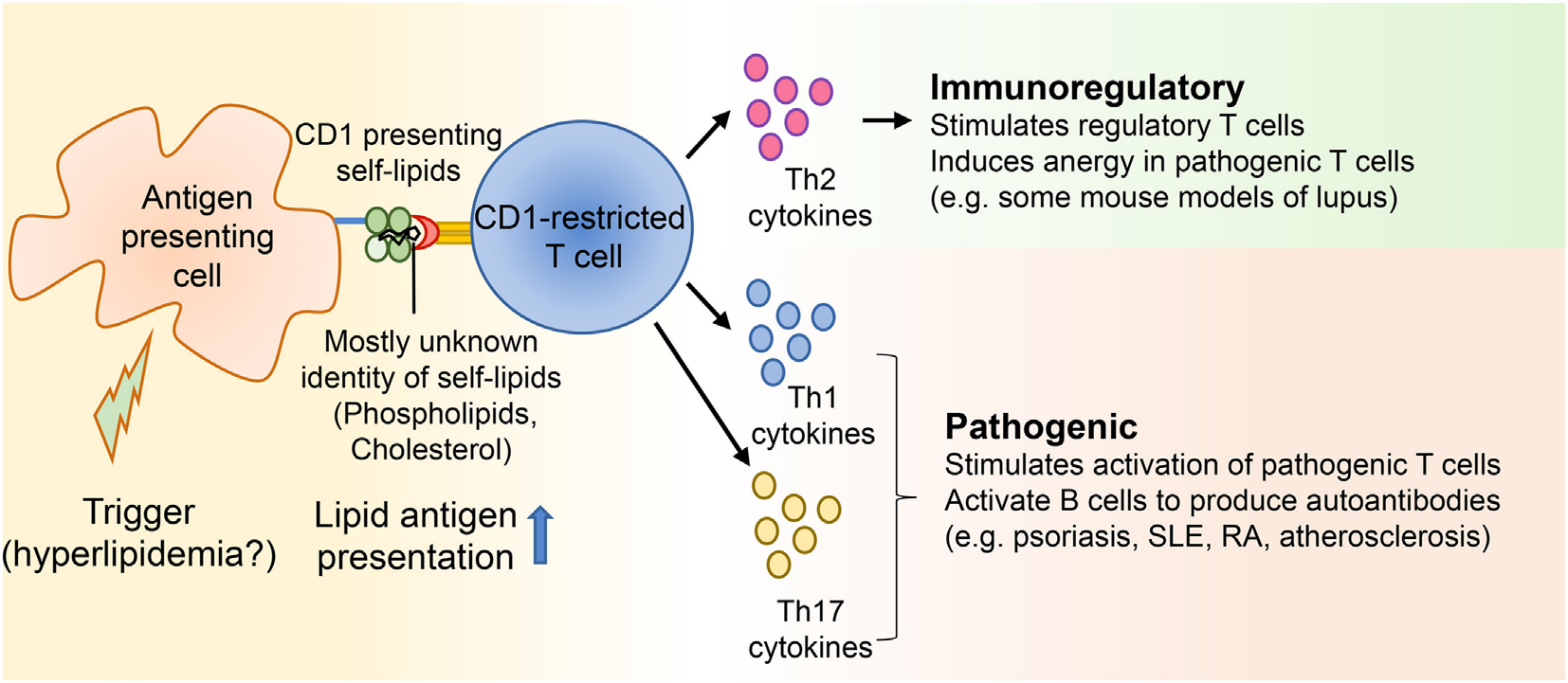
CD1-restricted T cells in hyperlipidemia associated autoimmune diseases. Autoimmune diseases develop as a result of genetic and environmental factors. Several studies suggest that some patients with diseases like systemic lupus erythematosus and psoriasis develop hyperlipidemia before onset of the autoimmune diseases. Yet, not many studies have directly demonstrated whether hyperlipidemia might be a trigger for certain autoimmune ailments. Since CD1 molecules present lipid antigens to T cells and a large portion of these T cells is autoreactive, self-lipid antigens can be presented to CD1-restricted T cells during autoimmune diseases, resulting in the secretion of Th1, Th2, and Th17 cytokines. These cytokines can then modulate either immunoregulatory or pathogenic immune responses by acting on both innate and adaptive immune players.

One of the major players of the adaptive immune system involved in the pathophysiology of autoimmune diseases is self-antigen reactive T cells (16). Even though most self-reactive T cells are eliminated in the thymus by the process of negative selection, some can escape. These T cells can recognize self-antigens in the periphery (16), so the autoantigens present within the affected tissues are thought to activate autoreactive T cells. Antigen-presenting cells like macrophages, dendritic cells, and B cells present the antigens to T cells, which secrete pro-inflammatory cytokines, leading to tissue damage (17). In the case of SLE, activated Th1 and Th2 effector T cells help B cells to produce autoantibodies (18). These antibodies can form immune complexes, which will then damage the kidney and lead to nephritis (19). In psoriasis, Th1-related cytokines like IFN-γ secreted by effector T cells are known to play a pathogenic role (20). Additionally, IL-17A is considered to be pathogenic in several autoimmune diseases and IL-17A blocking antibodies are currently used for treating autoimmune diseases like rheumatoid arthritis and psoriasis (20–22). Thus far, most studies have looked in to the role of peptide-specific autoreactive T cells in autoimmune disease initiation, progression, and maintenance. However, given that inflammation forms the crux of most autoimmune diseases, and that dyslipidemia is a potential trigger of chronic inflammation, it is imperative to uncover the role of lipid-specific autoreactive T cells in dyslipidemia-associated autoimmune diseases. Since lipid antigen-presenting molecules are widely expressed on a range of antigen-presenting cells in different tissues, it is conceivable that they contribute to the activation of cognate T cells when presented with lipids in inflammatory environments (Figure 1). Work by our lab and others have demonstrated a role for lipid-autoreactive T cells in psoriasis (23, 24). Thus, understanding the role of self-lipid reactive T cells in dyslipidemia-associated autoimmune diseases would not only lead to better treatment options for a myriad of these diseases but also allow for development of preventive measures to either delay or eliminate their progression. Thus, the subsequent sections of this review will focus on detailed discussions of lipid reactive T cells and their role in major dyslipidemia-associated autoimmune diseases like SLE, psoriasis, and RA, as well as its associated comorbidities such as atherosclerosis and obesity.

## CD1 Molecules

CD1 are a subset of MHC I-like molecules capable of presenting lipid antigens to T cells (25). Unlike MHC molecules, which are highly polymorphic, CD1 molecules exhibit very limited polymorphism, suggesting that antigens presented by each CD1 molecule are similar from one individual to the other (26). CD1 molecules are found in placental mammals and also birds, which indicates their ancient lineage (27). However, there are varying numbers and types of CD1 isoforms present in different animal species (28–32). CD1 molecules are divided into groups 1, 2, and 3 based on sequence homology and patterns of expression. Group 1 CD1 consists of CD1a, CD1b, and CD1c while CD1d is the sole group 2 CD1 molecule (33). While the abovementioned CD1 isoforms are expressed on the cell surface and present lipid antigens to T cells, the group 3 CD1, CD1e, acts as a chaperone in intracellular compartments to aid with antigen loading onto other CD1 molecules (34–36).

### CD1 Structure

*CD1* genes are paralogs of *MHC* genes and are unlinked from the *MHC* locus; genes encoding all CD1 isoforms are located on the long arm of chromosome 1q22-23 in humans (37–39). Like MHC I molecules, CD1 molecules form heterodimers of heavy α chains with β_2_ microglobulin (β_2_m), held together by non-covalent interactions (40–43). The antigen-binding grooves of CD1 molecules are usually narrower, deeper, and more voluminous than MHC I molecules and are lined with hydrophobic/neutral residues that facilitate binding of lipid molecules (44–48). This structural diversity allows CD1 isoforms to bind a range of different lipids, thus suggesting that each isoform may play a non-redundant role in the immune system.

### Antigens Presented by CD1 Molecules

Several studies have shown that CD1 molecules can present self-lipids to cognate T cells; yet, the physiological implication of self-lipid presentation under homeostatic and disease conditions remains unclear. We have recently shown that under conditions of hyperlipidemia, presentation of phospholipids and cholesterol by CD1b to cognate T cells drove the development of an inflammatory skin disease resembling psoriasis. In line with our findings, other groups have shown that CD1b can present phospholipids and GM1, a prototypic ganglioside, to T cells (49, 50). Apart from CD1b, CD1d is known to bind a range of glycosphingolipids and phospholipids (51–55). Interestingly, even though the antigen-binding groove of each CD1 molecule is unique, sulfatide, a sulfated glycolipid, is presented by all CD1 molecules, suggesting that each CD1 isoform is capable of presenting both shared and unique lipids (56). Additionally, CD1a can present the autoantigens phosphatidylcholine, lysophosphatidylcholine, and skin-derived apolar, headless oils (57, 58). CD1c can present a unique leukemia-associated methylated-lysophosphatidic acid and cholesteryl esters (59, 60). The ability of CD1 molecules to present such a diverse array of self lipids suggests their potential role in eliciting T cell responses under both steady state and pathological conditions.

### CD1 Expression and Tissue Distribution

In humans, CD1 molecules are distributed on a myriad of cell types and tissues. Both group 1 (CD1a, CD1b, and CD1c) and group 2 CD1d molecules are expressed on double-positive (CD4^+^CD8^+^) thymocytes (61). In peripheral tissues, group 1 CD1 molecules are expressed exclusively by professional antigen-presenting cells. Dendritic cell subsets from lymph node and skin can express any of the group 1 CD1 isoforms, while B cells can express CD1c (61–63). In contrast to group 1 CD1, group 2 CD1d expression is more widely distributed, found on both hematopoietic and non-hematopoietic-derived cells. Examples of CD1d-expressing cells include epithelial cells of the small bowel and colon, keratinocytes in skin, and hepatocytes in liver (61). CD1 expression can be altered in various autoimmune and inflammatory conditions, thus dictating the flavor of lipid-specific T cell responses. For example, CD1d was upregulated in the psoriatic plaques from patients with active psoriasis, while patients with active psoriasis and dyslipidemia exhibited increased CD1b expression in skin (23, 64). In contrast, CD1d expression was lower in B cells isolated from SLE patients compared to healthy controls, resulting in reduced ability to stimulate CD1d-restricted T cell cytokine production *ex vivo* (65). CD1 a, b, c, and d were shown to be upregulated in human atherosclerotic plaques compared to non-atherosclerotic arteries, leading to the potential for increased CD1-restricted T cell activation and inflammation (66). Whether CD1 molecules become up or downregulated in the context of autoimmunity and inflammation seems to be dependent on their specific environments and much is still to be learned. Many researchers utilize mice for immunological studies; however, mice only express CD1d and do not express group 1 CD1 molecules, limiting the ability to study CD1-restricted T cells in the context of autoimmunity (67). As a result, little is known about group 1 CD1-restricted T cells under normal and pathologic conditions leading to autoimmunity.

### Dysregulation in Antigen Presentation Under Dyslipidemia

Homeostatic presentation of lipid antigens by CD1 molecules can be disrupted by dyslipidemia. Cholesterol uptake and storage is a tightly regulated process that becomes dysregulated with genetic pre-dispositions and/or chronic overnutrition. Under steady-state conditions, cholesterol metabolism is regulated both intracellularly (SREBP2 pathway) and in circulation (LDL metabolism) (68). Dysregulated cholesterol metabolism within antigen-presenting cells has been linked to autoimmunity: Ito et al. reported that cholesterol accumulation within CD11c^+^ antigen-presenting cells drives autoreactive B cell and T cell expansion and promotes a lupus-like syndrome in mice (69). Our lab showed that serum from hyperlipidemic mice enhances IL-6 production by DCs, driving IL-17A production by autoreactive CD1b-restricted T cells in a model of psoriasis (23). Given that both group 1 and group 2 CD1 molecules are expressed by antigen-presenting cells and are upregulated in multiple autoimmune conditions, it will be important to further characterize the role that antigen-presenting cells play in autoimmunity and dyslipidemia in driving CD1-restricted T cell pathology.

## CD1-Restricted T Cells

### CD1d-Restricted Natural Killer T (NKT) Cells

CD1d-restricted NKT cells are divided into two main subsets, based on T cell receptor (TCR) usage. Type I NKT cells have an invariant TCR α chain (Vα14-Jα18 in mice and Vα24-Jα18 in humans) and thus are also referred to as invariant NKT (iNKT) cells (70–73). In mice, three β chains (Vβ7, Vβ8.2, and Vβ2) predominantly associate with the invariant α chain, while in humans, the invariant α chain pairs with Vβ11, an ortholog of the mouse Vβ8 (70–73). Unlike type I NKT cells, which have a semi-invariant TCR and recognize the marine sponge-derived lipid α-galactosylceramide (α-GalCer), type II NKT cells have diverse TCR usage and do not recognize α-GalCer (74–78). Rare populations of NKT cells (making up <1% of hematopoietic cells) have also been reported: NKT cells that use γδ chains for their TCR (primarily Vγ1.1 and Vδ6.3), and α-GalCer-reactive NKT cell population harboring a Vα10-Jα50 TCR were identified in mice (79, 80). Vα24^−^ CD1d-α-GalCer-specific T cells expressing CD4 or CD8αβ and using diverse Vα/Vβ chains have been reported in humans (78). NKT cells are “educated” in the thymus where CD1d-expressing cortical thymocytes mediate their positive selection as opposed to thymic epithelial cells that select conventional T cells (81). The lipid(s) responsible for the selection of NKT cells are largely unknown, though a recent study suggested that ether-linked lysophospahtidylethanolamine and lysophosphatidic acids might play a role in the thymic selection of iNKT cells (82).

In the thymus, NKT cells are characterized by the expression of the transcription factor PLZF (promyelocytic leukemia zinc finger protein), which is thought to impart the “innate-like” features to these T cells (83, 84). As NKT cells exit the thymus, they exhibit a pre-activated phenotype and have the capacity to rapidly produce Th1, Th2, and Th17 related cytokines upon TCR stimulation (85). These cytokines are produced when NKT cells interact with either self or foreign lipid antigens presented by the CD1d molecule. It is known that a small proportion of conventional T cells are self-peptide reactive. In contrast, most NKT cells can recognize self-lipid antigens, although the ability of self-lipids to stimulate cytokine production is dependent on two inputs: (1) the strength of TCR signaling and (2) the presence of cytokine driven co-stimulation (e.g., IL-12/IL-18 secreted by TLR-activated DCs) (86, 87). The nature of self-lipids that activate NKT cells during steady state and/or during a specific pathogenic challenge remain largely unknown. Thus, by virtue of their pre-activated status and their ability to be activated by self-antigens in the presence of the correct cytokine milieu, it is conceivable that NKT cells play an important role, either pathogenic or protective, in a range of infectious and autoimmune diseases as well as tumor immunity.

### Group 1 CD1-Restricted T Cells

In contrast to the copious amounts of information available on NKT cells, progress on group 1 CD1-restricted T cells is limited. Most studies have made use of long-term T cell clones isolated from patients infected with *Mycobacterium tuberculosis* and *Mycobacterium leprae* (88–94), though T cell clones derived from multiple sclerosis patients showing autoreactivity to several self glycosphingolipids have been described in the literature (49, 95). These T cell clones mostly have a diverse αβ TCR repertoire and can be CD4 or CD8 single positive or double negative, and capable of producing Th1, Th2, and/or Th17 related cytokines (88–94). While some of these T cell clones recognize lipid antigens from the mycobacterial cell wall, most CD1-restricted T cell clones described are autoreactive. In fact, the frequency of autoreactive group 1 CD1-restricted T cells makes up between 1 in 10 and 1 in 300 of all circulating T cells in humans, suggesting that they represent a substantial part of the T-cell repertoire in humans (96). Since most of the knowledge about these T cells comes from the study of T cell clones isolated from humans, the developmental program of group 1 CD1-restricted T cells and their physiologic responses during infection and autoimmunity are mostly unknown. Therefore, our lab generated a TCR transgenic mouse, expressing a CD1b-restricted self-lipid reactive TCR (HJ1Tg) and crossed it with mice co-expressing group 1 CD1b and CD1c (hCD1Tg) (97).

Characterization of HJ1Tg/hCD1Tg mice demonstrated that positive selection of autoreactive group 1 CD1-restricted T cells was mediated by thymocytes, similar to iNKT cells (97). CD1b-autoreactive HJ1 T cells were enriched in the liver and exhibited an activated/effector phenotype (CD44^hi^, CD69^+^, CD122^+^) in the naïve setting (97). Additionally, HJ1 T cells were shown to be protective against *Listeria monocytogenes* infection (97) and contribute to antitumor immunity against a CD1b^+^ T cell lymphoma (97, 98). In a recent study, we also demonstrated that under conditions of hyperlipidemia, HJ1 T cells contributed to the development of an inflammatory psoriasis-like skin inflammation (23). Although the nature of the lipid(s) recognized by HJ1 T cells in the context of *Listeria* infection remains unknown, HJ1 T cells in the context of hyperlipidemia were most likely activated by excess phospholipid and cholesterol species (23). These data suggest that while transient activation of group 1 CD1-autoreactive T cells may play a protective role in infections, chronic activation of autoreactive CD1-restricted T cells could lead to detrimental effects like initiation of inflammatory conditions and autoimmunity. A comparison of group 1 and group 2 CD1-restricted T cells is in Table 1.

**Table 1:** Comparison of group 1 and group 2 CD1-restricted autoreactive T cells.

	T cell receptor usage	Phenotype	Function	Self lipids recognized
Type I natural killer T (iNKT) cells (CD1d-restricted)	Vα14 Jα18 (mice)	Pre-activated CD4^+^/DN (mice), CD4^+^/CD8^+^/DN (human)	Th1, Th2, Th17, Tfh	Phospholipids, lysophospholipids, α-linked glycosphingolipids, plasmalogens (51, 53, 82)
	Vα24 Jα18 (humans) (70–73)			

Type II NKT cells (CD1d-restricted)	Diverse (76–78)	Pre-activated CD4^+^/DN (mice)	Th1, Th2	Phospholipids, lysophospholipids, β-linked glycosphingolipids, sulfatide (51, 54–56)

CD1a-restricted T cells	Diverse (24, 93)	Pre-activated CD45RO^+^, CLA^+^, CCR6^+^ (93, 94)	Th1, Th17, Th22	Squalene, free fatty acids, sulfatide (56–58)

CD1b-restricted T cells	Diverse	Pre-activated, CD4^+^/CD8^+^/DN	Th1, Th17	Phospholipids, GM1, sulfatide (49, 50, 56)

CD1c-restricted T cells	Diverse, αβ, or γδ T cells	Pre-activated, CD4^+^/CD8^+^/DN	Th1	Methylated lysophosphatidic acid (m-LPA), cholesteryl esters, phospholipids, sulfatide (56, 59, 60)

## The Role of CD1-Restricted T Cells in Autoimmune Diseases Correlated with Dyslipidemia

### Systemic Lupus Erythematosus

It has been well documented that atherosclerosis-related cardiovascular complications comprise the leading cause of death in SLE patients (2). Age, arterial hypertension, smoking habits, diabetes mellitus, obesity, and dyslipidemia are all known to be risk factors for the development of atherosclerosis-related cardiovascular diseases (CVD) among these patients (2). Dyslipidemia is specifically defined in SLE patients as increased total cholesterol, TG, LDL, and decreased levels of HDL (2). Studies have reported approximately 30% of SLE patients displaying signs of hyperlipidemia at the time of diagnosis, which increased to about 60% at 3 years post-diagnosis (99). Even though the mechanisms that cause hyperlipidemia in lupus patients is not well understood, some studies have suggested that the activity of enzyme lipoprotein lipase (LPL), which metabolizes lipids, is reduced and autoantibodies to LPL could be a reason for its reduced activity (100, 101). Like all autoimmune diseases, lupus is characterized by inflammation, often systemic. Under such conditions, LDL particles are more prone to oxidation (ox-LDL) (102). In fact, the process of atherosclerosis is initiated when macrophages ingest ox-LDL to become foam cells. Therefore, not surprisingly, anti-ox-LDL antibodies are present in lupus patients, contributing to the development of dyslipidemia-related cardiovascular ailments (103).

Since dyslipidemia and related pathologies are common in lupus patients and T cells are important players in lupus, exploring the role of lipid-reactive T cells is important. As iNKT cells are the most studied lipid-responsive T cell subset, copious amounts of information are available on these T cells in lupus, though some of the data are contradictory. When lupus was induced in C57BL/6 mice by injecting apoptotic cells, iNKT cells were activated and produced more IL-10 compared to IFN-γ; the absence of iNKT cells was associated with exacerbated disease as a result of increased autoantibody production and glomerular immune complex deposition (104). NKT cells also played a protective role when pristane, a hydrocarbon oil, was injected into BALB/c mice to stimulate a lupus-like disease. Absence of NKT cells (CD1d^−/−^ mice) in this model led to increased nephritis and serum autoantibodies (105). iNKT cell-specific cytokine production, IL-4, and TNF-α, were also decreased, with an expansion of marginal zone B cells (105). Interestingly, activation of iNKT cells with α-GalCer, upon induction of lupus using pristane, protected BALB/c mice from disease, but aggravated disease in SJL mice (106). As noted in the SJL mice, several studies have reported that iNKT cells in BWF1 mice induced autoantibody production from B cells, which promotes lupus pathogenesis (107, 108). In humans, CD1d expression on B cells and iNKT cell frequency and proliferative capacity generally decreases in lupus patients (65, 109, 110), suggesting a protective role for iNKT cell during lupus. However, another study showed that iNKT cells from SLE patients could induce CD1d-dependent CD40/CD40L-dependent anti-dsDNA antibody production by B cells, demonstrating a pathogenic role for iNKT cells in SLE (111). Aside from NKT cells, the role of other lipid-reactive T cells in SLE remains largely unexplored. One study showed that CD1c-restricted T cells isolated from SLE patients can promote class-switched IgG autoantibodies, mediated by CD1c, IL-4, and CD40 (112). Given that dyslipidemia is very prevalent in lupus patients, it is not surprising that lipid-specific autoreactive T cells may contribute to disease progression. However, due to the conflicting role of iNKT cells and the dearth of knowledge about other CD1-restricted T cells in lupus, more research needs to be conducted before their potential can be harnessed in the clinic.

### Psoriasis

Psoriasis, a primarily T cell driven autoimmune disease, which affects about 1–3% of the world’s population, is associated with hyperlipidemia (3, 113). Psoriatic patients also have a higher risk of developing cardiovascular disease such as atherosclerotic plaque formation (3). Additionally, psoriatic patients exhibit signs of systemic inflammation, which is largely mediated by neutrophils and T cells (20, 114). Although psoriatic patients are hyperlipidemic and psoriasis is a T cell driven disorder, the role of self-lipid reactive CD1-restricted T cells in psoriasis remains nebulous. Multiple studies have shown an increase in iNKT cells and CD1d expression within psoriatic lesions of mice and humans (64, 115, 116). Human skin engraftment of psoriatic and non-psoriatic skin onto SCID (severe combined immunodeficiency) mice has been the sole mouse model to study NKT cells in psoriasis; this model recapitulates a psoriasis-like disease in engrafted human skin when injected with psoriatic patient-derived lymphocytes (117, 118). An NKT cell line derived from a psoriatic patient was capable of producing IFN-γ when cocultured with CD1d-expressing keratinocytes and induced psoriasis plaque formation when injected into engrafted human skin SCID mice (64, 117). Since CD1d is expressed on keratinocytes and their expression is upregulated in psoriatic skin, it is thought that lipid antigens presented by these cells can activate NKT cells *in vivo* (61, 64).

Given the high frequency of autoreactive CD1-restricted T cells in humans and the presence of excess lipids under conditions of hyperlipidemia, it is surprising that the role of lipid-specific T cells remains understudied. Hyperlipidemia is induced in mice by either feeding them a high-fat diet, using mice that eat excessively due to genetic manipulations (obese mice) or knocking out genes important for lipid clearance from the blood. Recent work by our lab showed that upon induction of hyperlipidemia, CD1b-autoreactive T cells contributed to the development of psoriasis-like skin inflammation, characterized by a Th17 phenotype (23). Furthermore, there was preferential accumulation of phospholipids and cholesterol in the skin of these mice and the aforementioned lipids could be presented by CD1b to activate CD1b-autoreactive T cells. In humans, CD1b expression was increased in psoriatic compared to normal human skin and there were more CD1b-autoreactive T cells in the blood of affected individuals (23). Additionally, circulating CD1a-autoreactive T cell frequency increases in psoriasis patients compared to healthy controls (119). Presentation of self-lipids on CD1a drives the activation of cognate T cells with a Th17 effector phenotype. This results in the development of psoriatic-like skin inflammation (24, 119). Finally, headless apolar skin lipids can be presented by CD1a to CD1a-autoreactive T cells (58). It can be anticipated that some of these T cells might play a role in psoriasis, especially because they produce IL-17A and IL-22 in response to antigenic stimulation (58). A more in-depth examination of these T cells in psoriasis is warranted to determine the necessity of autoreactive CD1-restricted T cells in driving disease progression.

### Rheumatoid Arthritis

Rheumatoid arthritis is an autoimmune disease that affects the connective tissue of the synovial joints. It affects about 0.3–1% of the world’s population, with women being more susceptible than men (120). Interestingly, it is now clear that affected individuals have a 50% increased risk of developing CVD, even though levels of lipids associated with an increased risk of CVD, like cholesterol and LDL, do not always show an association with the development of RA (121–124). The mechanisms of RA disease development and progression are not fully understood; it is speculated that RA-associated inflammation consists of a proinflammatory milieu with self-reactive T and B cells potentially contributing to disease pathogenesis. Thus, it is conceivable that self-lipid reactive T cells might play a role in disease development. It has been demonstrated that iNKT cell number and function is altered in the peripheral blood of RA patients (125). Along with decreases in iNKT cell frequency, their capacity to secrete Th2-related cytokine secretions is also diminished. These findings suggest that iNKT cells may be involved in the disease process of RA, although further studies need to be completed to link these findings with physiological relevance (126). In collagen-induced arthritis (CIA) mouse models, which are commonly used as a model for RA, the absence of iNKT ameliorates disease severity (127, 128). Similar results were obtained when the interaction of CD1d with NKT cells was blocked by administration of anti-CD1d antibody (128). Interestingly, administration of OCH, a ligand known to skew iNKT cells to a Th2 type response, ameliorated disease in wildtype but not iNKT cell-deficient mice (129). Further, neutralization of IL-4 and IL-10 abrogated the OCH-mediated therapeutic benefit (129). Another mouse model of RA uses serum or immunoglobulins from K/BxN mice transferred to wild-type mice to promote joint inflammation. RA disease pathology score was reduced in NKT cell-deficient mice transferred with serum compared to wild-type mice (130). Additionally, iNKT cells were shown to infiltrate the joints of mice given K/BxN serum, secreting IFN-γ and IL-4, which inhibited anti-inflammatory TGF-β secretion in joint fluid (130). These studies show that CD1d-restricted NKT cells contribute to the pathogenesis in multiple mouse models of RA. However, the role of group 1 CD1-restricted T cells in RA remains unknown. Further, whether the role of self-lipid reactive T cells changes in this disease model under conditions of dyslipidemia needs to be elucidated.

### Obesity and Atherosclerosis

Due to the ease of tracking iNKT cells *in vivo*, it is no surprise that studies have examined the role of these T cells in high-fat diet fed, obese mice. In general, iNKT cells in adipose tissues have been implicated in maintaining immune homeostasis by producing IL-10, inducing an anti-inflammatory phenotype in macrophages (mediated by IL-4/STAT-6) and controlling the number and function of regulatory T cells (131). Recent studies have shown that CD1d expression and iNKT cell numbers decrease in obese humans and mice, leading to increased recruitment of proinflammatory macrophages in adipose tissues and insulin resistance (132–135). The cause of this decrease in iNKT cells is unclear, but insufficient stimulation of these T cells due to decreased CD1d expression could be a possibility. In contrast to the findings of the aforementioned studies, Wu et al. demonstrated that iNKT cells cause tissue inflammation in both high fat diet fed and obese mice (136). Further, adipocyte-specific CD1d deficiency ameliorated high fat diet-induced obesity and insulin resistance (137). A pathogenic contribution of iNKT cells under conditions of hyperlipidemia was also reported using β_2_m knockout mice injected with α-GalCer and fed a high fat diet. These mice had decreased inflammatory macrophage infiltration into adipose tissue compared to control C57BL/6 mice injected with α-GalCer and fed with a high fat diet (138). Since β_2_M knockout mice lack both CD8^+^ and NKT cells, the contribution of NKT cells alone to macrophage recruitment was not examined. Lastly, while one study has demonstrated a pathogenic role for type II NKT cells in high fat diet fed mice (139), no studies to date have explored group 1 CD1 expression and the role of group 1 CD1-restricted T cells in obesity.

Aside from diet-induced hyperlipidemia, knocking out genes important for lipid clearance like Apolipoprotein E (*Apoe*) and low density lipoprotein receptor (*Ldlr*) results in accumulation of lipids, characterized by increased serum cholesterol, TG and LDL with decreased HDL (140, 141). This lipid profile is similar to dyslipidemia observed in patients with autoimmune diseases. Traditionally these knockout mouse models have been used to study the formation of atherosclerotic plaques in murine blood vessels; unlike humans, mice do not naturally develop atherosclerotic plaques, even when put on high fat diet. Interestingly, in both *Apoe* and *Ldlr* knockout mice, a pathogenic role for iNKT cells has been reported. For example, CD1d deficiency significantly reduced atherosclerotic burden in atherosclerosis-prone mice (142–144). When mice were injected with α-GalCer, the process of atherosclerosis was accelerated and secretion of proatherogenic cytokines increased (142–144). Finally, it has been reported that iNKT cells play a pathogenic role during the initial phases of atherosclerotic plaque formation, but not during the later stages (143). Since atherosclerotic plaques harbor different species of oxidized and modified lipids, it is thought that CD1d-expressing DCs present these lipids to NKT cells, leading to their activation. This causes secretion of proinflammatory cytokines like IFN-γ, aggravating disease. A recent study showed that a monoclonal CD1b-autoreactive T cell did not significantly contribute to plaque formation in ApoE^−/−^ mice at later stages of disease (30-week-old mice) (23). Since it is now appreciated that immune cells are instrumental in the maintenance of atherosclerotic plaques, it is of interest to further explore the role of group 1 CD1-autoreactive T cells in this disease, especially because autoreactive group 1 CD1-restricted T cells form a large proportion of CD1-reactive T cells in humans, and group 1 CD1 molecules are expressed in human atherosclerotic plaques but not in normal arterial walls (66).

### The Effect of Statins, a Class of Lipid-Lowering Drugs, on Autoimmune Diseases

Statins, which are prescribed for lowering lipid levels in individuals with atherosclerosis, have favorable effects on autoimmune disease activity. In SLE patients, statin treatment was shown to lower proteinuria (145, 146). Additionally, statins can also have immunomodulatory functions such as increasing the presence of regulatory T cells, which are crucial for keeping autoimmune diseases at bay (147–149). Several studies have also noted a decrease in psoriasis area severity index scores in patients with psoriasis upon statin treatment (150, 151). However, another study did not find any statistically significant differences in psoriasis activity upon statin treatment (152). Therefore, it is too early to determine whether statins indeed ameliorate psoriasis. Finally, statin treatment has been shown to reduce disease activity scores of RA patients (153). These studies suggest a close link between lipid abnormalities and autoimmune diseases.

## Conclusion

Several autoimmune diseases have been strongly linked to lipid abnormalities (2). Whether dyslipidemia acts as a trigger for disease development or whether dyslipidemia is an outcome of autoimmune diseases is not well understood. In diseases like atherosclerosis and SLE, hyperlipidemia is known to develop before disease onset (2) and cause chronic inflammation (154). T cells play an important role in autoimmune disease progression; therefore, it is essential to decipher the role of T cells, specifically self-lipid reactive T cells, in dyslipidemia-associated autoimmune diseases. Current information on the role of these T cells in autoimmune diseases is shown in Table 2. As mentioned above, most of the knowledge comes from the study of iNKT cells. Even so, a slew of contradictory results has made it difficult to reach definitive conclusions about their role under conditions of dyslipidemia-associated autoimmunity. The conflicting results regarding the role of iNKT cells in various diseases may arise from a range of factors. For example, it is known that different subsets of iNKT cells have different functions (155). A majority of iNKT cells are CD4^+^ and can secrete IFN-γ and IL-4 under different stimuli (156). However, NK1.1^−^ iNKT cells are known to secrete more IL-17A (157). Additionally, the localization and microenvironment affect the function of iNKT cells. For example, adipose tissue-derived iNKT cells are known to play a regulatory role by not only producing IL-10 but also stimulating the activity of regulatory T cells (131). Furthermore, the genetic background of the mice can also affect iNKT function and thus their role in various disease conditions. It is known that iNKT cells from BALB/c mice secrete more IL-4 compared to IFN-γ than iNKT cells from C57BL/6 mice (155). Finally, microbiome compositions, which differ among animal housing facilities, may lead to discrepancies in results. Therefore, it is important to carefully evaluate these parameters before comparing results about iNKT cells in scientific studies. An additional parameter to note is that mouse models often do not fully mimic human autoimmunity. For example, mouse models of RA share autoreactive T cells, citrullinated autoantibodies, and macrophage/neutrophil infiltrate, but based on the method of disease induction, do not recapitulate the distribution of rheumatoid pannus and necessity of T cells as drivers of disease seen in humans (158). These reasons above highlight the importance of corroborating data from mouse models with human studies.

**Table 2:** Lipid-specific T cell involvement in autoimmune diseases and dyslipidemia.

Autoimmune disease	Type I NKT cells	Type II natural killer T cells	Group 1 CD1-restricted T cells
Atherosclerosis	Secrete proatherogenic cytokines, promote plaque formation in mice (142–144)	ND	ND
Systemic lupus erythematosus	Anti-inflammatory (65, 104–106, 109, 110) or pro-inflammatory role (106–108, 111) in mice and humans	ND	CD1c-restricted T cells promote class-switched IgG autoantibodies in humans (112)
Psoriasis	Increased frequency and CD1d expression in lesions of mice and humans (64, 115, 116)	ND	CD1b-autoreactive T cells drive skin inflammation *via* Th17 in HJ1Tg/hCD1Tg/ApoE^−/−^ mice (23), CD1a- and CD1b-autoreactive T cells increase in human skin/blood (23, 119)
Rheumatoid arthritis	Decreased frequency and Th2 cytokine production in blood (human) (125), promote inflammation (in mice collagen-induced arthritis and K/BxN model) (127–130)	ND	ND
Obesity	Anti-inflammatory role (in mice and humans) (132–135) or proinflammatory role (in HFD fed and db/db mice) (136)	Control visceral fat accumulation and insulin resistance in mice fed HFD (139)	ND

*ND, not determined*.

Aside from CD1d, the study of group 1 CD1-restricted T cells under conditions of dyslipidemia and autoimmunity remains obscure. However, recent studies from our lab demonstrated a pathogenic role of CD1b-autoreactive T cells in hyperlipidemia-associated psoriasis-like skin inflammation (23). Additionally, very little is known about CD1d-restricted type II NKT cells, owing to the lack of markers to track them *in vivo*. The presence of type II NKT cells in mice makes them more amenable than group 1 CD1-restricted T cells to study in murine mouse models; however, the differences in lipid metabolism between mice and humans makes it critical to corroborate any murine findings of type II NKT cells with human studies. It is important to study these T cell subsets in more depth not only because they form a substantial proportion of the total human T cell population but also because a majority of these T cells are known to be autoreactive.

Though the role of iNKT cells has been deciphered in some autoimmune diseases, the identity of self-lipids recognized by these T cells remain largely unknown. The general thought is that antigen-presenting cells expressing CD1-self-lipid complexes activate CD1-restricted T cells. Activated T cells produce cytokines that are either immunomodulatory or inflammatory. The nature of the cytokines secreted is influenced by the functional state of the antigen-presenting cells, the type of self-lipid being presented, and the microenvironment. It is crucial to identify these lipids not only for tracking these T cells *in vivo*, but also for harnessing their potential in the clinic. Additionally, very few studies have examined the role of CD1-restricted T cells under dyslipidemia and autoimmune disorders. Thus, the study of self-lipid reactive T cells in this context is still in its infancy; more research investigating the links between autoimmunity, inflammation, and dyslipidemia could inform both diagnosis and treatment of autoimmune diseases in the future.

## Author Contributions

All authors listed have made a substantial, direct, and intellectual contribution to the work, and approved it for publication.

## Conflict of Interest Statement

The authors declare that the research was conducted in the absence of any commercial or financial relationships that could be construed as a potential conflict of interest.
